# 

**Published:** 1868-04-15

**Authors:** 


*CONTENTS:*

Experimental Medicine.— Experimental Researches into the subject of the action of Phosphorus upon the living Tissues. By Walter Hay, M.D., Chicago, Ill. .......... 251
A Case of Shoulder Presentation. By B. F. Ross, M.D., South Pass, Ill. 258
Reply to a case of Instrumental Delivery. By E. K. Travers, M.D. Amboy, Ill. .......... 260
Foreign Correspondence — Letter from Paris .......... 262
A Case of Accidental Hæmorrhage. By De Laskie Miller, M.D., Prof. in Rush Medical College .......... 268
Ocluded Anus. By H. A. Chase, M.D., Viroqua, Wis. .......... 270

EDITORIAL:

News Items .......... 271
Indiana State Medical Society .......... 272
A Card .......... 272
University of Michigan. The Bribe Accepted .......... 272
Fluid Extracts .......... 272

LOOT:

Milk for Babes .......... 276
Decolorizing Tinct. Iodine .......... 278
Treatment of Venereal Diseases in Paris .......... 279
Perserving Anatomical Specimens .......... 280
American Medical Association .......... 280
Receipts since January 1st .......... 282

